# Evaluation of pathogenicity of *Botrytis* species isolated from different legumes

**DOI:** 10.3389/fpls.2023.1069126

**Published:** 2023-03-27

**Authors:** Elīna Brauna-Morževska, Frederick L. Stoddard, Biruta Bankina, Jānis Kaņeps, Gunita Bimšteine, Irina Petrova, Ingrīda Neusa-Luca, Ance Roga, Dāvids Fridmanis

**Affiliations:** ^1^ Institute of Soil and Plant Sciences, Latvia University of Life Sciences and Technologies, Jelgava, Latvia; ^2^ Viikki Plant Science Centre, and Helsinki Sustainability Science Centre, Department of Agricultural Sciences, University of Helsinki, Helsinki, Finland; ^3^ Human Genetics and Disease Mechanisms, Latvian Biomedical Research and Study Centre, Riga, Latvia

**Keywords:** *Botrytis*, legumes, chocolate spot, gray mold, pathogenicity tests, *B. euroamericana*, *B. medusae*

## Abstract

Fungi of genus *Botrytis* are important pathogens of legumes, causing gray mold and chocolate spot diseases. The use of molecular methods to identify pathogens has resulted in the discovery of several new *Botrytis* species and new associations of pathogens with diseases. Thus, chocolate spot of faba bean is now associated with at least four species: *B. fabae*, *B. cinerea*, *B. pseudocinerea* and *B. fabiopsis*. Species of *Botrytis* differ in host plant, pathogenicity, fungicide resistance and other relevant properties that affect disease control. The aim of this study was to identify the species of *Botrytis* isolated from different legume crops and to evaluate their *in vitro* pathogenicity. Between 2014 and 2019, 278 isolates of *Botrytis* were obtained from infected legumes in Latvia. A phylogenetic analysis was carried out by sequencing three nuclear genes, RPB2, HSP60, and G3PDH, considered to be diagnostic for species in this genus. A set of 21 representative isolates was selected for pathogenicity tests on detached leaves of faba bean, field pea, lupin and soybean using 5-mm mycelium-agar plugs. The diameter of the formed lesions under the inoculated plug was measured crosswise each day. The datasets were subjected to analysis of variance with the split-plot design of the experiment and repeated-measures model. Six species were identified: *B. cinerea*, *B. fabae*, *B. pseudocinerea*, *B. fabiopsis*, *B. euroamericana* and *B. medusae*. In addition to the expected combinations of host and pathogen, naturally occurring infections of *B. fabiopsis* were found on chickpea, *B. euroamericana* on faba bean and *B. medusae* in lupin seeds. Species and isolate had significant effects on pathogenicity on all crops tested. Several isolates were pathogenic on two or more host species: two of *B. pseudocinerea*, two of *B. cinerea*, two of *B. fabiopsis* and the one of *B. medusae*. One isolate of *B. pseudocinerea* and two of *B. fabiopsis* caused primary lesions on all five host species. The results show that these *Botrytis* species have a broad host range that should be borne in mind when planning crop sequences and rotations.

## 1 Introduction

Legume crops provide various benefits for cropping systems and are regaining an important role in European agriculture. Between 2009-2014 and 2015-2020 the area for production of dry pulses and protein crops increased by 64.5% in the European Union. In the same period in the Baltic countries, areas sown to grain legumes increased an average of five times (from 5820 to 42250 ha in Latvia, 51850 to 181090 ha in Lithuania and 10730 to 48600 ha in Estonia) (Eurostat, 2021).

In Latvia, the important grain legume crops are faba bean (*Vicia faba* L.) and field pea (*Pisum sativum* L.). Lupins (*Lupinus angustifolius* L. and *L. luteus* L.), common vetch (*Vicia sativa* L.), soybean (*Glycine max* (L.) Merr.) and common bean (*Phaseolus vulgaris* L.) are grown on small areas (Sterna et al., 2020; Central Statistical Bureau of Latvia, 2022). Lucerne (*Medicago sativa* L.), clovers (*Trifolium* spp.), fodder galega (*Galega orientalis* Lam.), and white sweet clover (*Melilotus albus* Medik.) are legumes grown for forage (Līpenīte and Kārkliņš, 2015). Given this diversity of legumes and their uses in agriculture, it is important to know about their shared diseases.

Chocolate spot disease of faba bean has usually been attributed to *Botrytis fabae* and to a lesser extent to *B. cinerea* (Davidson et al., 2007; Elad et al., 2016). Distinguishing species by the symptoms they cause is usually not possible, considering that species can occur in complexes. In 2010 in China, a new *Botrytis* sp., named *B. fabiopsis*, was found and described in complex with *B. cinerea* and *B. fabae* in faba bean where it occurred with higher frequency than *B. fabae* (Zhang et al., 2010). *B. pseudocinerea* was first discovered in French vineyards, then isolated from faba bean in Germany, and increasing numbers of studies have revealed its presence on different host-plants in Europe, North America, China and Australia (Walker et al., 2011; Saito et al., 2014; Harper et al., 2019; Xue et al., 2019; Plesken et al., 2021). These four *Botrytis* species have all been associated with the chocolate spot disease of faba bean, most recently in Latvia (Bankina et al., 2021).

Recent studies have shown the ability of the lesser known *Botrytis* species to infect different legumes *in vitro*. *B. euroamericana* was able to infect chickpea, field pea, lentil and faba bean leaves during pathogenicity tests *in vitro* at the same rate as *B. cinerea* (Moparthi et al., 2023). *B. caroliniana*, *B. pyriformis*, *B. aclada* and *B. paeoniae* formed lesions on faba bean leaves *in vitro*, but have not been isolated from field-grown faba bean (Li et al., 2012; Zhang et al., 2016; Garfinkel et al., 2017).

The species of *Botrytis* mainly have narrow host-plant specificity, but there are several species that infect a wide range of host plants. *B. cinerea* as a generalist can infect over 1400 plant species, including many legume crops (Ma & Michailides, 2005; Elad et al., 2016; Garfinkel, 2021). *B. pseudocinerea* has been found on several host plants, including grapevine, strawberry and oilseed rape, and was the predominant pathogen isolated from a faba bean field in Germany (Walker et al., 2011; Plesken et al., 2015; Weber and Hahn, 2019; Xue et al., 2019). The role of *B. pseudocinerea* in other legumes is unclear yet, but the species has a worldwide distribution and it seems to have a wide host range.

The minimum recommended interval for legumes in a crop rotation is 3-4 years (Pande et al., 2009). According to the ability of several *Botrytis* species to infect different legumes, we may suggest that *Botrytis* spp. can transfer between host-plants, volunteer plants and alternate hosts. Thus, for the successful inclusion of legume crops in agriculture, it is necessary to identify the *Botrytis* species occurring in legumes and to evaluate their pathogenicity for each legume crop. Exploring the interaction between *Botrytis* species and legume hosts will provide important information for understanding disease control methods in legumes and possible sources of inoculum.

Hence, we set out to identify the range of *Botrytis* species that could be isolated from grain legume crops in Latvia and to test their pathogenicity on other legume crop species. In this way, we could determine the potential for cross-infection of legume crops from other legumes or their post-harvest residues.

## 2 Materials and methods

### 2.1 Sample collection and fungal isolation

**Figure 1:**
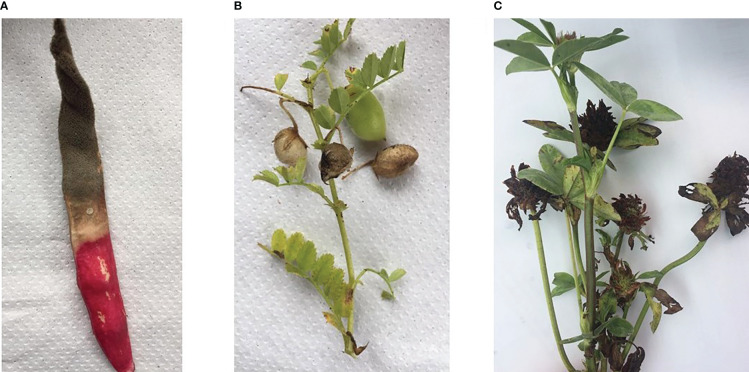
Legume crops with visible fungal infection symptoms: common bean pod **(A)**, chickpea pod **(B)** and red clover flowers **(C)**.

Infected plant parts of different legumes were collected between 2014 and 2019 (**Figure 1**; **Table 1**). Small tissue parts of infected leaves, pods and flowers or whole seeds were surface sterilized for 1 min with 1% sodium hypochlorite, rinsed three times in sterile distilled water and placed onto potato-dextrose agar (PDA) with streptomycin (100 mg L^-1^) and penicillin (100 mg L^-1^). Cultures were incubated at 20°C under 12 h light and 12 h darkness for one week. Single-hyphal isolates were obtained from each colony and grown on PDA (Leyronas et al., 2012).

**Table 1:** Identities, origins and GenBank accession numbers of the sequences for the selected *Botrytis* spp. isolates.

Species name	Isolate name	Host plant	Infected part	Year of isolation	RPB2	HSP60	G3PDH
*B. cinerea*	17B10-1	Faba bean	Leaf	2014	OP623535	OP623556	OP623577
*B. cinerea*	18B3	Faba bean	Seed	2016	OP623539	OP623560	OP623581
*B. cinerea*	19B009	Common bean	Pod	2019	OP623527	OP623548	OP623569
*B. cinerea*	19B014	Red clover	Flower	2019	OP623543	OP623564	OP623585
*B. cinerea*	19B026	Chickpea	Pod	2019	OP623529	OP623550	OP623571
*B. cinerea*	19B048	Narrow-leafed lupin	Stem	2019	OP623531	OP623552	OP623573
*B. euroamericana*	19B053-3	Faba bean	Leaf	2019	OP623532	OP623553	OP623574
*B. fabae*	17B28	Faba bean	Leaf	2016	OP623537	OP623558	OP623579
*B. fabae*	17B4	Faba bean	Leaf	2016	OP623534	OP623555	OP623576
*B. fabae*	19B053-4	Faba bean	Leaf	2019	OP623533	OP623554	OP623575
*B. fabiopsis*	18B16	Faba bean	Seed	2016	OP623546	OP623567	OP623588
*B. fabiopsis*	17F4	Faba bean	Seed	2016	OP623538	OP623559	OP623580
*B. fabiopsis*	17F9	Faba bean	Seed	2016	OP623544	OP623565	OP623586
*B. fabiopsis*	19B024	Chickpea	Leaf	2019	OP623528	OP623549	OP623570
*B. fabiopsis*	17B24	Faba bean	Seed	2016	OP623536	OP623557	OP623578
*B. fabiopsis*	B11	Faba bean	Leaf	2016	OP623542	OP623563	OP623584
*B. fabiopsis*	19B175	Faba bean	Leaf	2019	OP623526	OP623547	OP623568
*B. medusae*	19B047	Narrow-leafed lupin	Seed	2019	OP623530	OP623551	OP623572
*B. pseudocinerea*	18B2-3	Faba bean	Seed	2016	OP623545	OP623566	OP623587
*B. pseudocinerea*	18B11	Faba bean	Seed	2016	OP623541	OP623562	OP623583
*B. pseudocinerea*	18B6	Faba bean	Leaf	2016	OP623540	OP623561	OP623582

Pure cultures were evaluated for morphological characteristics and subjected to preliminary molecular genetic analyses of the ITS region, using ITS1-F, ITS4 (reverse) primers. The final collection comprised 278 *Botrytis* putative isolates showing traits typical of the genus. After the identification to species level by sequencing three nuclear genes RPB2, HSP60 and G3PDH (described in the following section), a set of 21 isolate was selected for further investigations. Isolates of all determined species originated from different hosts were choosen.

For each selected isolate, 10 working cultures were established by transferring a mycelial plug (5 mm ø) from the margins of the one-week-old culture: nine for pathogenicity tests and one for molecular genetic analyses.

### 2.2 Molecular genetic analyses

About 10 mg of fungal material was collected from petri dishes using a sterile cell scraper with a 20 mm lifter blade (Bellco Glass, USA) (in our experience this amount covers ~ 1/3 of scraper blade) and suspended in 500 µl NucleoMag^®^ 96 Plant kit (Macherey-Nagel, Germany) Lysis buffer. This was followed by homogenization for 2×60 sec using FastPrep^®^-24 instrument and Lysing Matrix D (MP Biomedicals, USA), phenol (Alfa Aesar, Germany) - chloroform (Merck, USA) treatment and subsequent purification with the NucleoMag^®^ 96 Plant kit according to the manufacturer’s instructions.

The identification to species level was carried out by sequencing three nuclear genes: RNA polymerase II (RPB2), Heat shock protein 60 (HSP60) and Glyceraldehyde 3-phosphate dehydrogenase (G3PDH). Primer combinations (**Table 2**) were used according to Staats et al. (2005), and Raja et al. (2017) who showed that these three gene sequences were diagnostic for discriminating *Botrytis* species from each other.

**Table 2:** Legume crops used in the pathogenicity tests.

Crop	Latin name	Cultivars
Faba bean	*Vicia faba*	‘Lielplatone’, ‘Laura’
Field pea	*Pisum sativum*	‘Doloresa’, ‘Florida’
Narrow-leafed lupin	*Lupinus angustifolius*	‘Probor’, ‘Sonet’
Yellow lupin	*Lupinus luteus*	‘Lejaskurzeme’
Soybean	*Glycine max*	‘Laulema’, ‘Skulptor’

The amplification of RPB2, HSP60 and G3PDH by PCR was carried out for 40 cycles (98°C-5 sec, 59°C-5 sec, 72°C-20 sec, GeneAmp PCR System 9700 (Applied Biosystems, USA)) in a total volume of 20 µl. The reaction mixture was comprised of following components: 10 µl of H2X Phire Plant Direct PCR Master Mix (Thermo Fisher Scientific, USA), 0.3 µM forward primer and 0.3 µM reverse primer (**Table 3**) (Staats et al., 2005) and eluted 1 µl of fungal DNA solution. The success of the amplification was verified through the inspection of PCR products by agarose gel electrophoresis. Samples were considered positive when they contained a >700bp PCR fragment.

**Table 3:** Primers for PCR amplification and sequencing.

Gene	Forward primer (for1)	Reverse primer (rev1)
G3PDH	5’-ATTGACATCGTCGCTGTCAACGA-3’	5’-ACCCCACTCGTTGTCGTACCA-3’
HSP60	5’-CAACAATTGAGATTTGCCCACAAG-3’	5’-GATGGATCCAGTGGTACCGAGCAT-3’
RPB2	5’-GATGATCGTGATCATTTCGG-3’	5’-CCCATAGCTTGCTTACCCAT-3’

Positive reaction mixtures were cleaned up from excess of dNTPs and primers through employment of Exonuclease I (0.5 µl) (Thermo Fisher Scientific, USA) and Shrimp Alkaline Phosphatase (2 µl) (Thermo Fisher Scientific, USA) (incubated for 40 min at 37°C and inactivated at 95°C for 20 min). Further, 1 µl of cleaned fragment solution was transferred to BigDye^®^ Terminator v3.1 Cycle Sequencing reaction mixture which was prepared according to the manufacturer’s instructions (Applied Biosystems, USA). Both DNA strands of every PCR product were sequenced using primers ITS1-F, ITS4 (reverse), G3PDHfor1, G3PDHrev1, HSP60for1, HSP60rev1, RPB2for1 and RPB2rev1 and sequencing products were analyzed on 3130xl Genetic Analyzer (Applied Biosystems, USA). The ITS region sequences from each isolate were aligned using MEGA11 software (v. 11.0.13) and the created contig was BLAST^®^ searched against the NCBI nucleotide database to identify taxonomic sources of records with the highest sequence identity (https://blast.ncbi.nlm.nih.gov/Blast.cgi).

G3PDH, HSP60, and RPB2 gene sequences were aligned with other *Botrytis* sequences, downloaded from NCBI nucleotide database, including the sequences of *Sclerotinia sclerotiorum* and *Monilinia fructigena* as outgroup taxa (Staats et al., 2005). The alignment of sequences was carried out by Multiple Alignment using Fast Fourier Transform (MAFFT) (Madeira et al., 2022), alignments were trimmed by trimAl software employing the strict method (Capella-Gutiérrez et al., 2009) and clustered by MEGA5 software employing the ‘Neighbor Joining’ algorithm to construct the phylogenetic tree (Tamura et al., 2011).

### 2.3 Pathogenicity test

The test of pathogenicity was performed *in vitro* on leaves of two cultivars of each of faba bean, field pea, narrow-leafed lupin and soybean, and one of yellow lupin (**Table 2**).

Two layers of sterile filter paper sheets were used to line metal trays (40 × 30 × 5 cm). Four 28 cm long glass sticks were arranged into each tray to support the leaves. An equal amount of water was poured into each tray, so that it was moist but water did not accumulate in puddles. Leaves were obtained from the middle level of plants at BBCH growth stage 35 (5 visibly extended internodes) and rinsed twice with sterile water. Whole leaves of faba bean and field pea were put onto the glass sticks in the metal trays so that only the petiole touched the wet filter paper. Soybean leaves were divided into leaflets and placed in metal trays so that only the cut surface touched the water. For lupin leaves, three glass sticks were tied together and three middle leaflets were detached and placed on them so as not to touch the filter paper.

Each tray contained 30 plant leaflets, one for each of the isolates and some controls. Agar plugs with *Botrytis* isolates (5 mm ø) were placed on leaflets with the mycelium side down. Two non-inoculated agar plugs were used in each tray as a control. After inoculation, the trays were covered with plastic film to maintain high humidity and incubated at 20°C in the dark for 72 h. The diameter of the formed lesions under the inoculated plug was measured crosswise once a day until some of lesions reached the edge of the leaf. Four replicates were prepared and the experiment was repeated, giving 8 replicates. A further repeat was conducted for narrow-leafed lupin ‘Probor’, giving 12 replicates. Mean lesion diameter was considered as the measure of pathogenicity.

### 2.4 Data analysis

Leaflet datasets were subjected to analysis of variance using SPSS v. 27 (IBM Inc., Chicago, IL, USA) within each host genus (yellow and narrow-leafed lupins were analysed together). A simple split-plot model was applied to the data from each day, where the culture box was the main plot and the leaflet was the subplot. The repeated-measures model was then applied to 4 days of data from faba bean and soybean as well as to 3 days of data from lupins and pea. The output was corrected to take into account the split-plot design of the experiment.

## 3 Results

### 3.1 Molecular-genetic analysis

**Figure 2:**
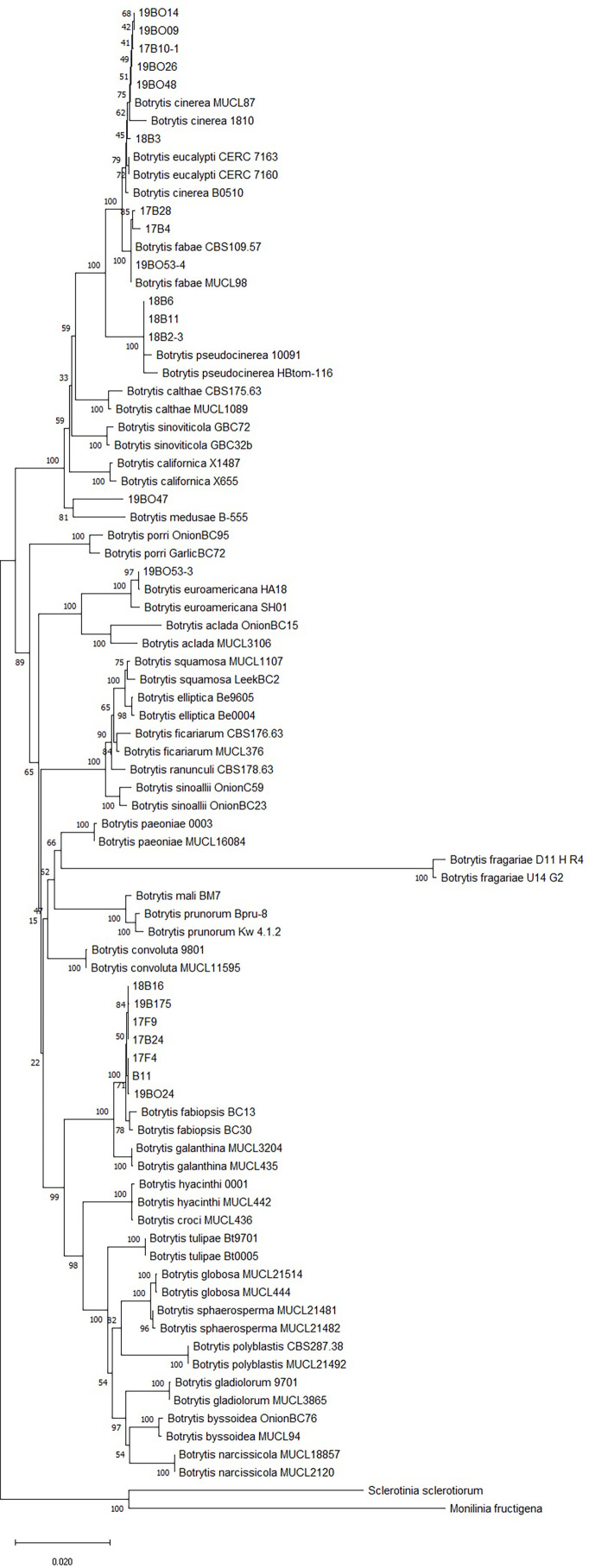
Neighbor joining tree of RPB2, HSP60 and G3PDH gene sequences. The tree describes the relationship of *Botrytis* species isolates collected from legumes in Latvia to named *Botrytis* species including the sequences of *Sclerotinia sclerotiorum* and *Monilinia fructigena* as outgroup taxa. Branches with <50% bootstrap support are not shown.

The 26 isolates collected from legumes in this study with the *Botrytis* spp. isolates from GenBank formed a clade with 99% bootstrap support, distinct from the closely related *M. fructigena* and *S. sclerotiorum* outgroups. Branches with under 50% bootstrap support were not included in the phylogenetic tree (**Figure 2**). Phylogenetic analysis of the combined gene datasets confirmed that all isolates belonged to the genus *Botrytis*. Six isolates clustered with *B. cinerea* references, three with *B. fabae*, seven with *B. fabiopsis* and three with *B. pseudocinerea*. One isolate was grouped with *B. medusae* and one isolate with *B. euroamericana* (**Table 1**). Five isolates did not clearly group with identifiable clades, so they were not considered further in this study.

### 3.2 Pathogenicity tests

The first symptoms of infection were observed 24 h after inoculation of all host cultivars. The main effects of *Botrytis* species and isolates were significant for all measures on all host species (**Table 4**). The effects of replicate and of the day × replicate interaction were significant for pea, because lesion diameters were 1 mm greater in replicates 1 to 4 than those in replicates 5 to 8 on day 1, which continued until day 3, when it was no longer significant.

**Table 4:** Summary analysis of variance of *Botrytis* spp. pathogenicity on faba bean, pea, lupin and soybean leaves.

	Faba bean		Pea		Lupin		Soybean	
Source of variation	df	Mean Square	df	Mean Square	df	Mean Square	df	Mean Square
Cultivar	1	0.404	1	24.561 **	2	5.878	1	30.782 *
Replicate	7	2.654	7	8.333 *	11	5.188	7	7.740
Box stratum residual	7	1.645	7	1.451	14	4.373	7	3.179
Species	5	22.065 ***	5	22.112 ***	5	74.368 ***	5	22.787 ***
Cultivar * Species	5	0.290	5	1.679	10	5.584 **	5	2.107 *
Isolate	15	9.450 ***	15	22.596 ***	15	30.975 ***	15	17.385 ***
Cultivar * Isolate	15	1.859	15	3.635 ***	30	5.234 ***	15	2.688 ***
Leaflet stratum residual	270	1.319	269	1.114	357	1.996	279	0.834
Days	3	56.387 ***	2	127.395 ***	2	175.465 ***	3	30.732 ***
Days * Cultivar	3	0.298	2	0.112	4	3.807 **	3	6.278 ***
Days * Replicate	21	0.239	14	0.522 *	22	1.054	21	0.171
Days * Box stratum residual	21	0.233	14	0.155	28	0.654	21	0.260
Days * Species	15	2.751 ***	10	0.590 ***	10	6.876 ***	15	1.273 ***
Days * Cultivar * Species	15	0.166	10	0.608 ***	20	0.823 ***	15	0.355 ***
Days * Isolate	45	1.030 ***	30	1.447 ***	30	4.184 ***	45	1.176 ***
Days * Cultivar * Isolate	45	0.209	30	0.614 ***	60	0.690 ***	45	0.417 ***
Days x leaflet stratum residual	810	0.216	538	0.121	714	0.299	837	0.108

df, degrees of freedom; *** p<0.001; ** p<0.01; * p<0.05

Differences between cultivars were significant in soybean, where lesions were larger on ‘Skulptor’ than those on ‘Laulema’ from day 2 onwards, and in pea, where the lesions on ‘Doloresa’ were larger than those on ‘Florida’ on all dates. The cultivar × species interaction was significant in soybean and lupin, while the cultivar × isolate interaction was significant for all species except faba bean. On days 1 and 2, *B. cinerea* and *B. pseudocinerea* caused much larger lesions on the yellow lupin ‘Lejaskurzeme’ than on the narrow-leafed cultivars. On day 1, lesions of *B. medusae* on ‘Probor’ were much larger than those on the other two lupin cultivars and on days 2 and 3, ‘Sonet’ had smaller lesions than the other two cultivars. On days 2 and 3, both *B. euroamericana* and *B. fabae* produced larger lesions on ‘Sonet’ than on the other two lupins.

To compare the pathogenicity of *Botrytis* spp., the optimal assessment day was examined for each host species, when the leaves were still fresh and the lesion diameter was determined. For pea, the optimal assessment day was the third, for lupins the second, for soybean the fourth, and for faba bean the fifth day after infection. Based on the size of the lesions and the characteristics of spot formation in each host species, we divided the pathogenicity of the isolates into three classes: – low (primary lesion only), moderate, and high (**Table 5**).

**Table 5:** Pathogenicity of *Botrytis* spp. on faba bean, lupin, pea and soybean leaves.

Species	Isolate	Lesion size on faba bean leaves at Day 5, mm			Lesion size on pea leaves at Day 3, mm			Lesion size on lupin leaves at Day 2, mm			Lesion size on soybean leaves at Day 4, mm		
		Mean		Std. Error	Mean		Std. Error	Mean		Std. Error	Mean		Std. Error
*B. cinerea*	17B10-1	0.0	^e^	1.2	1.9	^g^	0.9	4.6	^de^	0.8	1.1	^de^	0.8
*B. cinerea*	18B3	5.4	^bcde^	1.2	4.2	^efg^	0.9	1.8	^ef^	0.8	0.4	^de^	0.8
*B. cinerea*	19B009	1.1	^de^	1.2	10.5	^bc^	1.0	11.0	^ab^	0.8	6.7	^ab^	0.9
*B. cinerea*	19B014	0.1	^e^	1.2	9.4	^bcd^	0.9	11.0	^ab^	0.8	2.6	^cde^	0.8
*B. cinerea*	19B026	3.4	^cde^	1.2	10.5	^bc^	0.9	6.9	^cd^	0.8	3.5	^abcde^	0.8
*B. cinerea*	19B048	4.2	^cde^	1.2	10.7	^bc^	0.9	6.4	^cd^	0.8	2.7	^bcde^	0.8
*B. euroamericana*	19B053-3	1.3	^cde^	1.3	5.2	^defg^	0.9	1.8	^ef^	0.8	0.2	^de^	0.8
*B. fabae*	17B28	13.5	^a^	1.4	4.0	^efg^	0.9	0.1	^e^	0.8	0.3	^de^	0.8
*B. fabae*	17B4	3.8	^cde^	1.3	4.3	^efg^	0.9	1.3	^ef^	0.8	0.4	^de^	0.8
*B. fabae*	19B053-4	4.6	^cde^	1.3	2.3	^fg^	0.9	0.3	^e^	0.8	0.1	^e^	0.8
*B. fabiopsis*	17B24	3.9	^cde^	1.2	2.2	^fg^	0.9	0.6	^e^	0.8	0.4	^de^	0.8
*B. fabiopsis*	17F4	11.7	^ab^	1.2	16.5	^a^	1.0	9.7	^abc^	0.8	5.8	^ab^	0.8
*B. fabiopsis*	17F9	2.5	^cde^	1.2	3.0	^fg^	0.9	1.0	^ef^	0.8	1.2	^de^	0.8
*B. fabiopsis*	18B16	6.1	^bcde^	1.3	1.9	^g^	0.9	0.9	^ef^	0.8	0.8	^de^	0.8
*B. fabiopsis*	19B024	4.7	^cde^	1.2	2.9	^fg^	0.9	0.5	^e^	0.8	0.1	^e^	0.8
*B. fabiopsis*	19B175	5.8	^bcde^	1.4	10.0	^bc^	0.9	7.6	^bcd^	0.8	7.0	^ab^	0.8
*B. fabiopsis*	B11	2.3	^cde^	1.3	2.7	^fg^	0.9	0.6	^ef^	0.8	0.8	^de^	0.8
*B. medusae*	19B047	0.0	^e^	1.2	8.4	^bcde^	0.9	11.9	^a^	0.8	0.3	^de^	0.8
*B. pseudocinerea*	18B11	7.1	^abcd^	1.2	6.7	^cdef^	0.9	10.2	^abc^	0.8	4.5	^abcd^	0.8
*B. pseudocinerea*	18B2-3	7.7	^abc^	1.3	13.0	^ab^	1.0	6.9	^cd^	0.8	7.2	^a^	0.8
*B. pseudocinerea*	18B6	1.2	^de^	1.2	1.0	^g^	0.9	0.5	^e^	0.8	0.1	^e^	0.8

Means followed by same letter within each column are not significantly different according to Tukey’s HSD. Shading colors relatively divide the pathogenicity of isolates into three classes. Green shading indicates primary lesions and low pathogenicity, amber shading moderate pathogenicity and red shading high pathogenicity.

**Figure 3:**
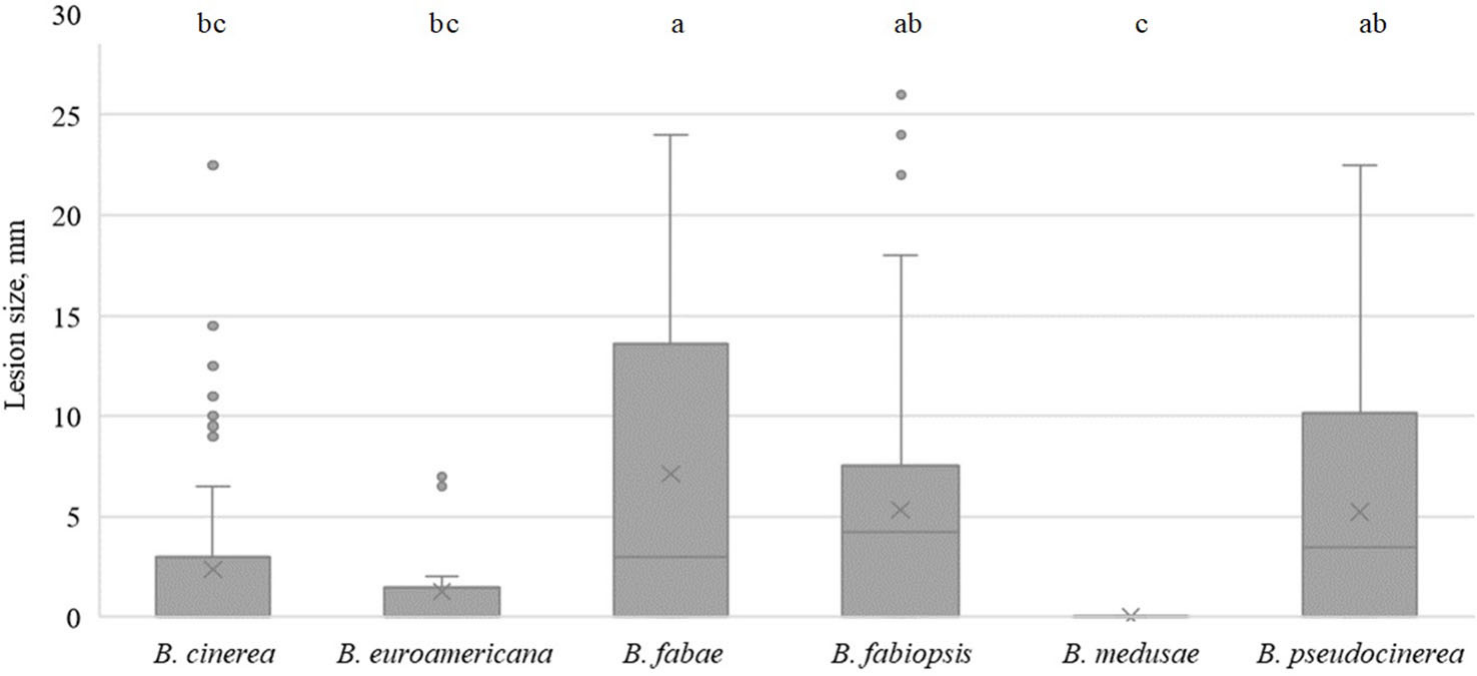
Box-plot diagram of the lesion diameters on faba bean leaves five days after inoculation with isolates of 6 *Botrytis* species. Boxes with the same letter are not significantly different according to Tukey’s HSD.

In faba bean, *B. fabae* caused the largest lesions on day 5 (**Figure 3**), with isolate 17B28 being the strongest (**Table 5**). The lesions produced by *B. pseudocinerea* and *B. fabiopsis* were somewhat smaller than those caused by *B. fabae* and were similar to each other. *B. fabiopsis* isolate 17F4 produced lesions second to those of *B. fabae* 17B28 in size. Isolates 18B11 and 18B2-3 of *B. cinerea* exhibited high pathogenicity on faba bean leaves; however, as a species it was less pathogenic than *B. pseudocinerea*. Lesions caused by *B. euroamericana* were observed only in cv. ‘Lielplatone’. *B. medusae* did not cause any injury in either faba bean cultivar.

**Figure 4:**
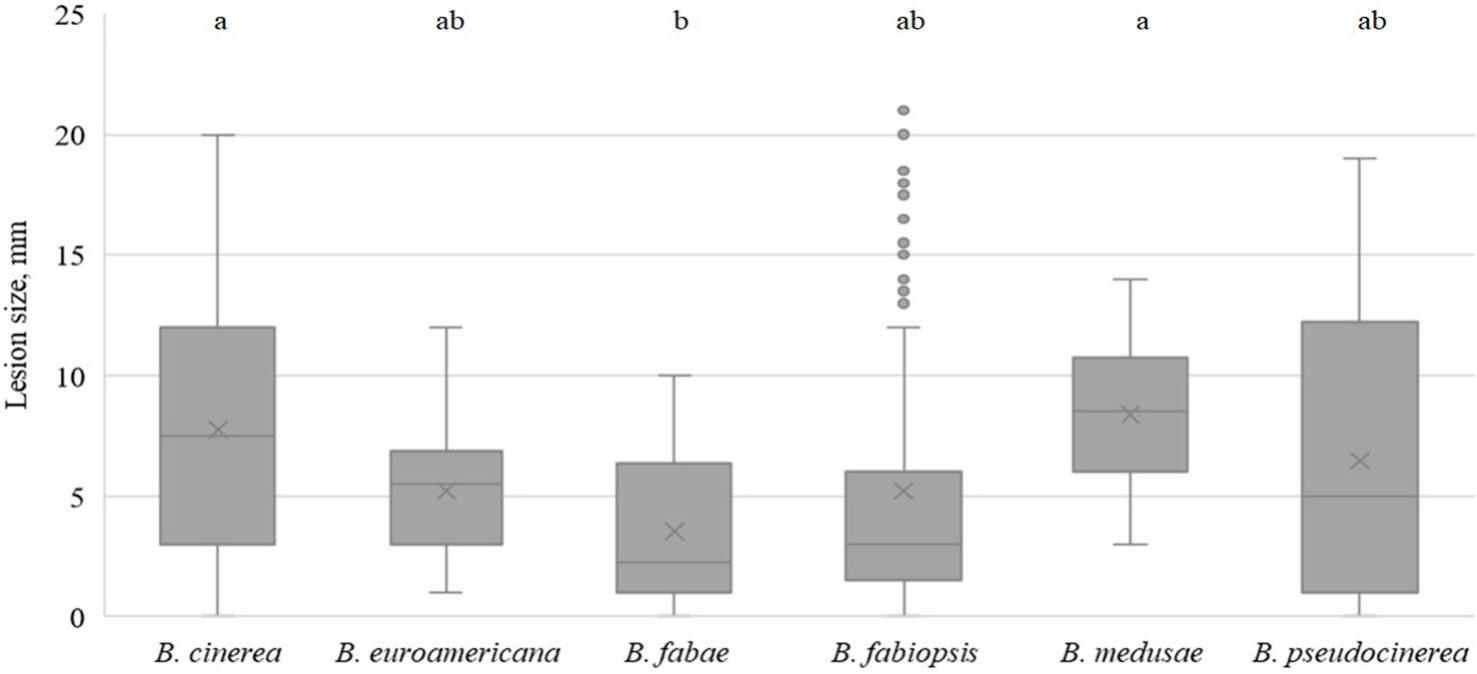
Box-plot diagram of the lesion diameters on field pea leaves three days after inoculation with isolates of 6 *Botrytis* species. Boxes with the same letter are not significantly different according to Tukey’s HSD.

On day 3, all species caused lesions on pea leaves, and the most pathogenic were *B. medusae* and *B. cinerea* (**Figure 4**). *B. fabae* was significantly less pathogenic. In general, *B. fabiopsis* caused primary or moderate lesions, except 17F4 and 19B175 (**Table 4**), which formed large lesions on both the tested pea cultivars. Isolate 17F4 caused lesions that were even larger than those of the most pathogenic *B. cinerea* isolates. *B. pseudocinerea* isolate 18B2-3 was also responsible for large lesions.

**Figure 5:**
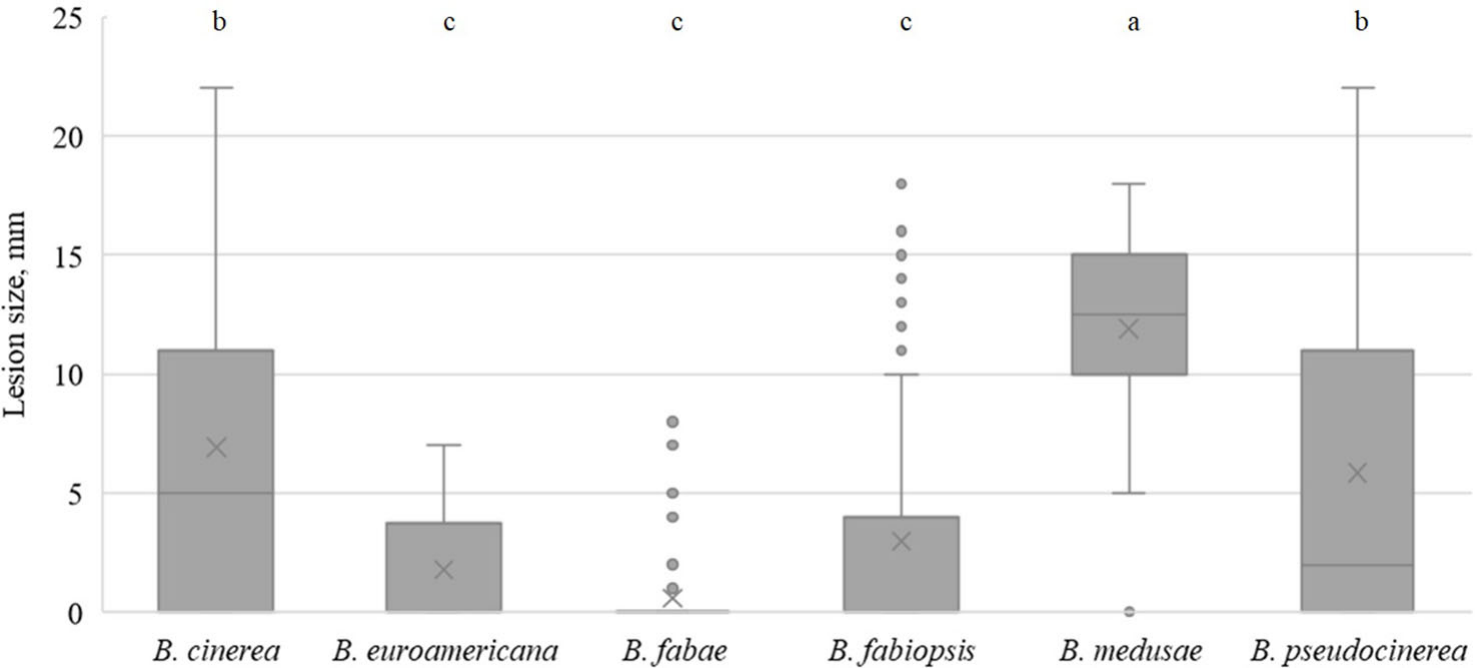
Box-plot diagram of the lesion diameters on lupin leaves two days after inoculation with isolates of 6 *Botrytis* species. Boxes with the same letter are not significantly different according to Tukey’s HSD.

On lupin leaves, *B. medusae* was the most pathogenic (**Table 5**) and caused significantly larger lesions than the other *Botrytis* spp. (**Figure 5**). *B. pseudocinerea* and *B. cinerea* caused moderate to large lesions, depending on the isolate, and their pathogenicity was significantly higher than that of *B. fabae*, *B. fabiopsis* and *B. euroamericana*, although *B. fabiopsis* isolate 17F4 produced lesions that were nearly as large as those of the most pathogenic *B. cinerea* isolates (**Table 5**).

**Figure 6:**
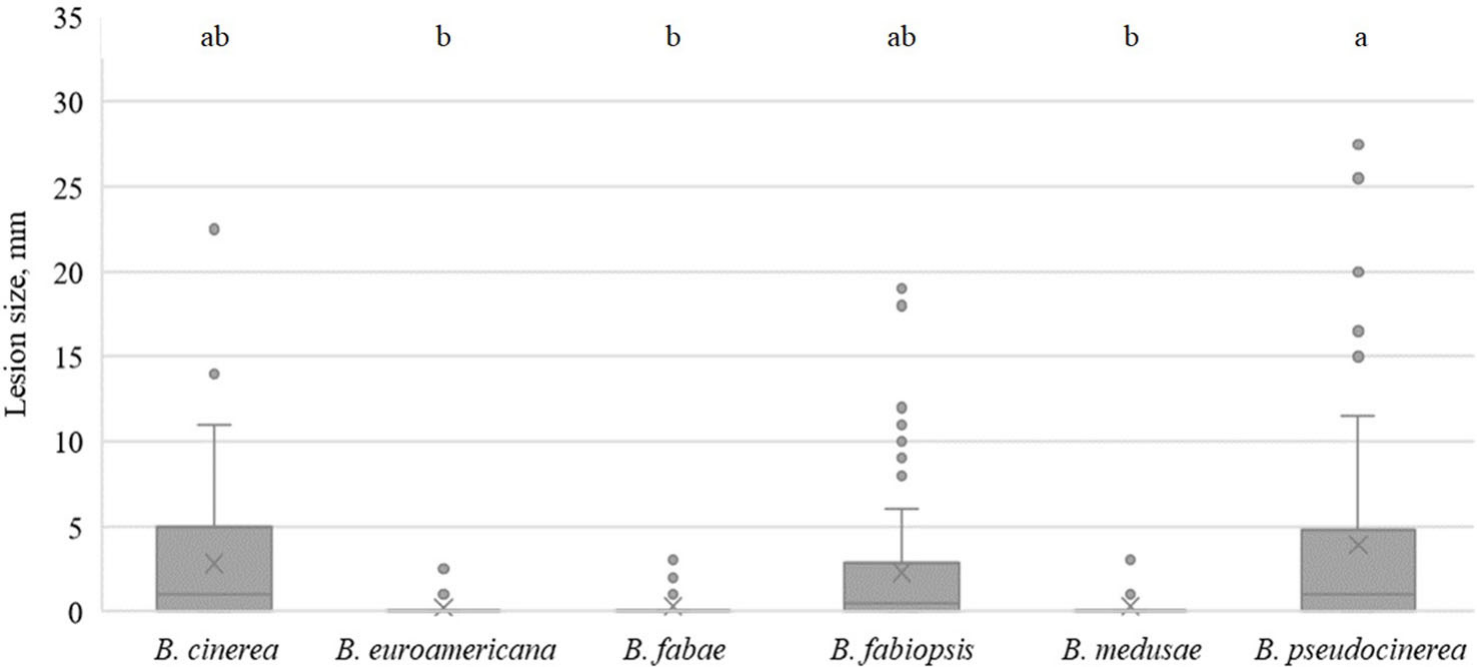
Box-plot diagram of the lesion diameter on soybean leaves four days after inoculation with isolates of 6 *Botrytis* species. Boxes with the same letter are not significantly different according to Tukey’s HSD.

Lesion sizes were smaller in soybean than on the other host species (**Figure 6**). *B. fabae*, *B. euroamericana* and *B. medusae* caused only primary lesions on both soybean cultivars. One *B. cinerea*, two *B. fabiopsis* and one *B. pseudocinerea* isolate caused lesions larger than 5.5 mm in diameter (**Table 5**), that larger than those caused by the agar plug of inoculum. Cultivar ‘Skulptor’ was notably more susceptible to *B. pseudocinerea* than cultivar ‘Laulema’.

Certain isolates were highly pathogenic to all host species; notably *B. fabiopsis* 17F4 (highly pathogenic on all), *B. cinerea* 19B009 and *B. pseudocinerea* 18B2-3 (highly pathogenic on three and moderately pathogenic on one), and *B. fabiopsis* 19B175 and *B. pseudocinerea* 18B11 (highly pathogenic on two and moderately pathogenic on two host species (**Table 5**)). At the other extreme, *B. fabiopsis* 17F9 and B11, along with *B. pseudocinerea* 18B6 had, low pathogenicity in all hosts.

## 4 Discussion

A high diversity of *Botrytis* spp. was recovered from these legumes. In the main legume crop of Latvia, faba bean, five *Botrytis* spp. were isolated: *B. cinerea*, *B. fabae*, *B. pseudocinerea*, *B. fabiopsis* and *B. euroamericana*.

*B. fabae* is considered the most important pathogen and primary causal agent of chocolate spot disease in faba bean because of its narrow specialization (Davidson et al., 2007). In the present study, *B. fabae* caused lesions on faba bean and moderate lesions on pea, but only primary lesions on soybean and lupin, thus acting as a narrowly specialized faba bean pathogen. *B. fabae* has occasionally been isolated from other genera in the *Fabaceae*, including field pea (Elad et al., 2016).

*B. cinerea* can be observed as a pathogen of faba bean, either separately or in a complex with *B. fabae* (Harrison, 1988; Zhang et al., 2010). As expected, *B. cinerea* was isolated from a wide variety of legumes including faba bean, common bean, red clover, chickpea, and narrow-leafed lupin. Pathogenicity tests did not exhibit clear regularity between the natural host-plants and pathogenicity of these isolates. Isolate 17B10-1 from faba bean did not produce any lesions on faba bean during these tests, while isolate 19B048 from narrow-leafed lupin caused more damage to pea than to lupin. These results suggest that these isolates were saprophytic on plant tissues that were already suffering from the attack of other pathogens. The *Botrytis* genus is known to have high intraspecific variation, and unstable pathogenicity has been observed within individual isolates (Garfinkel, 2021). In general *B. cinerea* exhibited a high variability in pathogenicity.

In this study, some isolates of *B. pseudocinerea* and *B. fabiopsis* produced large lesions on the detached leaves of all host species and some caused mild disease. These species have been much less studied than the previous two and found in relatively few countries. Both have been implicated in the chocolate spot disease of faba bean in several countries, including Latvia (Bankina et al., 2021). *B. pseudocinerea* has been found in various unrelated host plants (Plesken et al., 2015; Garfinkel, 2021).

*B. fabiopsis* was isolated from faba bean leaves and seeds as well as from chickpea leaves. Most isolates acted as a narrow specialist pathogen; however, but one isolate was highly pathogenic in all hosts. The current study is the first report of *B. fabiopsis* naturally occurring in chickpea and the first demonstration that it can cause disease in pea, narrow-leafed lupin, yellow lupin and soybean. To date, no information is available on the occurrence of *B. fabiopsis* elsewhere than in faba bean in China (Zhang et al., 2010) and Latvia (Bankina et al., 2021).

The isolate of *B. euroamericana* was obtained from faba bean leaves but caused only primary lesions on this species, while it was moderately pathogenic on pea leaves. The present isolate was purified from two *B. cinerea* isolates obtained from a single leaf lesion, so it may have been an endophytic or saprophytic follower on the lesion caused by *B. cinerea*. This is the first report of *B. euroamericana* occurring naturally on faba bean leaves. Originally, *B. euroamericana* was isolated from peony and grapes in North America and Europe (Garfinkel et al., 2017). Later *B. euroamericana* was reported as a causative agent of grey mold on chickpea leaves in the USA (Moparthi et al., 2020) and was observed on lentil and dry pea seeds. The ability to cause lesions in several hosts from different plant families during pathogenicity tests confirms the wide host range of this pathogen.

The single *B. medusae* isolate was obtained from lupin seeds and of all the *Botrytis* species tested here, it showed the highest pathogenicity on lupin leaves, along with convincing pathogenicity on field pea leaves while it formed primary lesions on faba bean leaves. To the best of our knowledge, this study is the first report to *B. medusae* occurring naturally in lupin. Originally, *B. medusae* was collected in 2015 from wine grapes (Harper et al., 2019) and has otherwise been little reported on.

Garfinkel's (2021) review suggested that many of the newly discovered *Botrytis* species may be polyphagous. This study confirmed the occurrence of four new species, discovered since 2010, on crops different from the original host-plant families: *B. fabiopsis* on chickpea, *B. pseudocinerea*, *B. euroamericana* on faba bean, and *B. medusae* on narrow-leafed lupin.

Pathogenicity tests on detached leaves do not always reflect the true pathogenicity of a pathogen under field conditions; however, if there is a possibility that isolates in nature may behave similar to the pathogenicity tests, the risk of transmission between host plants may be higher. Further research of lesser-known *Botrytis* sp. in different legumes is needed to clarify their occurrence and significance in disease initiation in all hosts.

## Data availability statement

The original contributions presented in the study are included in the article/supplementary material, further inquiries can be directed to the corresponding author/s.

## Author contributions

EB-M and FS performed the data analysis and wrote the manuscript. EB-M and IP performed pathogenicity studies. EB-M and JK did the sampling and genetic characterization of the isolates. BB and GB did the conception and design of the study. IN-L did fungal isolation and creation of fungal collection. AR and DF performed sequencing. All authors contributed to the article and approved the submitted version.
